# The future for genetic studies in reproduction

**DOI:** 10.1093/molehr/gat058

**Published:** 2013-08-26

**Authors:** G.W. Montgomery, K.T. Zondervan, D.R. Nyholt

**Affiliations:** 1 Department of Genetics and Computational Biology, Queensland Institute of Medical Research, Brisbane, Australia; 2 Genetic and Genomic Epidemiology Unit, Wellcome Trust Centre for Human Genetics, University of Oxford, Oxford, UK; 3 Nuffield Department of Obstetrics and Gynaecology, John Radcliffe Hospital, University of Oxford, Oxford, UK

**Keywords:** reproductive traits, GWAS, gene discovery, translation, review

## Abstract

Genetic factors contribute to risk of many common diseases affecting reproduction and fertility. In recent years, methods for genome-wide association studies (GWAS) have revolutionized gene discovery for common traits and diseases. Results of GWAS are documented in the Catalog of Published Genome-Wide Association Studies at the National Human Genome Research Institute and report over 70 publications for 32 traits and diseases associated with reproduction. These include endometriosis, uterine fibroids, age at menarche and age at menopause. Results that pass appropriate stringent levels of significance are generally well replicated in independent studies. Examples of genetic variation affecting twinning rate, infertility, endometriosis and age at menarche demonstrate that the spectrum of disease-related variants for reproductive traits is similar to most other common diseases. GWAS ‘hits’ provide novel insights into biological pathways and the translational value of these studies lies in discovery of novel gene targets for biomarkers, drug development and greater understanding of environmental factors contributing to disease risk. Results also show that genetic data can help define sub-types of disease and co-morbidity with other traits and diseases. To date, many studies on reproductive traits have used relatively small samples. Future genetic marker studies in large samples with detailed phenotypic and clinical information will yield new insights into disease risk, disease classification and co-morbidity for many diseases associated with reproduction and infertility.

## Introduction

Genetic inheritance influences risk for many reproductive traits and diseases. Over the last 20 years, the genes responsible for many rare reproductive disorders with Mendelian patterns of inheritance have been identified by genetic linkage mapping and positional cloning in families with multiple affected individuals (Botstein and Risch, 2003). For example, mutations in at least 20 genes cause hypogonadotropic hypogonadism including Kallmann syndrome and mutations in 14 genes cause gonadal failure associated with hypergonadotropic hypogonadism (Layman, 2013). Many of these mutations are rare in the general population, as they change protein function and generally result in large increases in disease risk for mutation carriers, an important reason why linkage studies in families have generally been very successful in their identification.

Genetic factors also contribute to risk of many common traits and diseases like endometriosis and uterine fibroids that have a more complex aetiology involving both genetic and environmental factors. In contrast to genetic mutations in Mendelian traits, genetic variants involved in complex diseases confer a modest increase in susceptibility rather than a large increase in risk. Common genetic variants in complex disease are more amenable to detection using population-based association (typically case–control) studies, rather than family-based linkage designs. Population-based association studies include hypothesis-based ‘candidate gene’ and hypothesis-free ‘genome-wide association study (GWAS)’ designs. Although there is a large literature reporting candidate gene associations, most results have not been validated in large independent studies (Montgomery *et al.*, 2008; Stranger *et al.*, 2011; Rahmioglu *et al.*, 2012). Methods for GWAS developed and applied in the last 5 years have revolutionized gene-mapping studies in complex traits and identified a large number of gene regions with strong evidence for association with many diseases (Hindorff *et al.*, 2009; Stranger *et al.*, 2011; Visscher *et al.*, 2012). This approach has been applied to reproductive traits and diseases and novel gene regions affecting risk reported for endometriosis, uterine fibroids, age at menarche, age at menopause and cancers of the reproductive tract. In general, the effects of individual associations are small, and for each disease the cumulative effect of all associations thus far accounts for only a small proportion of the predicted genetic variation (Visscher *et al.*, 2012).

There is ongoing debate about the value and application of results from GWAS (Stranger *et al.*, 2011; Fugger *et al.*, 2012; Marian, 2012; Visscher *et al.*, 2012). Results that pass appropriate stringent levels of significance are generally well replicated in independent studies. They provide novel insights into biological pathways contributing to disease risk (Stranger *et al.*, 2011; Fugger *et al.*, 2012; Visscher *et al.*, 2012) and suggest new drug treatments (Sanseau *et al.*, 2012). In most cases, these results represent a starting point to define genes and pathways contributing to disease risk and many more risk genes are still to be discovered. There remains an important role for larger genetic studies for many reproductive traits and diseases and from genotyping samples with more detailed information on risk factors and clinical presentation. Future directions will include analysis of rare coding variants and functional annotation of variants in regulatory regions through continuing advances in genetics and genomics. The GWAS results also provide valuable datasets to evaluate genetic contributions to disease severity, sub-classes of related diseases and co-morbidity between diseases (Painter *et al.*, 2011; Stranger *et al.*, 2011; Visscher *et al.*, 2012; Lee *et al.*, 2013).

## Genome-wide association studies

GWAS methods provide a powerful approach for mapping disease genes. The techniques have developed from spectacular advances in genotyping technology, greater understanding of the structure of common variants in the human genome, and continued advances in computing power and software tools for analysis of large datasets (Stranger *et al.*, 2011; Bochud, 2012; Visscher *et al.*, 2012). More than 30 million SNPs segregate in human populations. Genotyping all common variants remains a major task, but it has been shown by the International HapMap Consortium project (Consortium, 2005) that most of the common variation can be captured by genotyping a representative set of SNPs chosen to ‘tag’ common variants using array-based techniques (see below). Typical GWAS projects that genotype ∼500 000 tagSNPs in several thousand cases and controls to test for association with disease will capture most of the common variation with minor allele frequencies >10%, but very dense marker sets must be typed to capture all variation.

Studies should be carefully designed taking account of the characteristics of the disease or trait being studied. One important consideration is to ensure cases and controls are well matched for ethnicity to reduce the chances of false-positive association signals caused by differences in allele frequency where one ethnic group is over-represented in either the case or the control group. Analytical methods such as principal component analysis can be used to adjust for some unmeasured population differences (population stratification) (Stranger *et al.*, 2011) by identifying outliers or including principal components in the analysis. Family-based designs for association provide an alternative to case–control studies (Laird and Lange, 2006; Benyamin *et al.*, 2009). They have advantages for quality control of genotype data and overcoming issues of population stratification. Family studies generally have lower power than case–control designs when genotyping equivalent numbers of individuals, but can include analyses that are not possible with unrelated individuals such as evaluation of imprinted genes or combined linkage and association analysis.

Genetic association studies type many markers and conduct multiple tests for marker trait associations. Results from GWAS have clearly demonstrated the need to adequately correct for the multiple testing and to replicate results in independent samples before reporting association between genetic markers and common disease traits. To identify associations with a genome-wide false-positive probability around 5% (i.e. an overall *P*-value of 0.05 taking account of all independent statistical tests conducted), a stringent threshold must be set for each individual SNP-disease association test to guard against reporting false-positive associations. For genome-wide tests of association this is usually set at *P* < 5 × 10^−8^ (Dudbridge and Gusnanto, 2008). Genome-wide significant results that meet these requirements generally show replication in subsequent studies and in some cases across ethnic groups.

## Reproductive traits

Gene discovery using GWAS methods is documented in the Catalog of Published Genome-Wide Association Studies at the National Human Genome Research Institute (http://www.genome.gov/gwastudies/) (Hindorff *et al.*, 2009). The current catalogue (May 2013) includes 1604 publications and association results for 10127 SNPs. The list of traits associated with reproduction includes studies on age at menarche, age at menopause, endometriosis, uterine fibroids, cancers of the reproductive tract and response to drug treatments (Table I). These data present evidence for genome-wide significant associations for 32 traits reported in 71 publications. Results for individual studies can be viewed through the catalogue and in the primary research publications. The number and statistical significance of risk loci identified for reproductive traits are relatively similar to results from GWAS of other traits and some examples described below illustrate how the novel gene discoveries provide important insights into the genetic architecture of complex diseases.

**Table I:** Traits and diseases associated with reproduction included in the Catalog of Published Genome-Wide Association Studies at the National Human Genome Research Institute^a^ (http://www.genome.gov/gwastudies/) (Hindorff *et al.*, 2009).

Disease or trait	Publications	Significant associations	Suggestive associations
Adverse response to aromatase inhibitors	1	0	1
Breast cancer	18	21	25
Breast cancer (survival)	1	0	2
Endometrial cancer	1	1	1
Endometriosis	4	8	7
Erectile dysfunction	1	1	5
Erectile dysfunction and prostate cancer treatment	1	1	22
Estradiol levels	1	0	8
Hypospadias	1	1	0
Male fertility	0	0	0
Male infertility	1	0	5
Menarche (age at onset)	2	34	9
Menarche and menopause (age at onset)	1	6	0
Menopause (age at onset)	3	19	14
Ovarian cancer	3	3	7
Ovarian reserve	1	0	7
Polycystic ovary syndrome	2	14	0
Pre-eclampsia	0	0	0
Premature ovarian failure	1	0	1
Prostate cancer	13	49	19
Prostate cancer (gene × gene interaction)	1	0	36
Prostate cancer mortality	0	0	0
Prostate-specific antigen levels	3	10	0
Response to tamoxifen in breast cancer	1	0	1
Sex hormone-binding globulin levels	1	10	8
Sexual dysfunction (female)	1	0	3
Sexual dysfunction (SSRI/SNRI related)	1	0	5
Testicular cancer	2	2	0
Testicular germ cell cancer	1	6	0
Testicular germ cell tumour	1	3	2
Testosterone levels	2	1	5
Uterine fibroids	1	3	2

Data are included as significant associations if SNPs had *P*-values <5 × 10^−8^ and as suggestive associations if SNPs had *P*-values <10^−5^. Publications were included if they reported at least one significant association and loci were counted once even if also reported in subsequent papers.

^a^Data accessed on 22/05/13.

### Endometriosis

**Figure 1:**
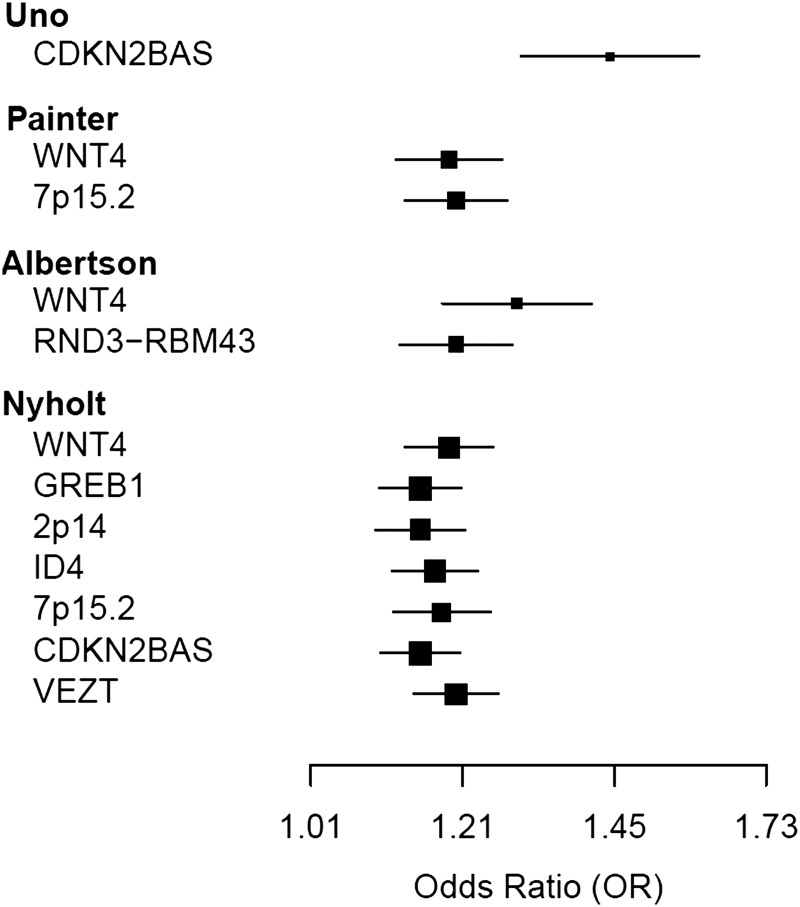
The effect size estimates for genome-wide significant results reported in GWAS studies in endometriosis. The estimated effect sizes (per allele change) are represented by box and whisker plots for the sentinel SNP (SNP with the lowest *P*-value) for each genome-wide significant locus. The mid-point of each box represents the point effect estimate (OR) for each locus. The width of the line shows the 95% confidence intervals of the effect estimate of individual studies. The first three studies are results from individual GWAS papers (Uno *et al.*, 2010; Painter *et al.*, 2011; Albertsen *et al.*, 2013) and the fourth study is a meta-analysis of GWAS data from the first two studies (Nyholt *et al.*, 2012).

Endometriosis is a common gynaecological disease associated with severe pelvic pain and subfertility. Disease risk is influenced by both genetic and environmental factors and the heritability is estimated at 51% (Treloar *et al.*, 1999). Four GWAS have been published for endometriosis, two in Japanese populations (Adachi *et al.*, 2010; Uno *et al.*, 2010) and two in populations of European descent (Painter *et al.*, 2011; Albertsen *et al.*, 2013). Genome-wide significant signals were reported in three of these studies (Fig. 1). The first study by Uno and others (Uno *et al.*, 2010) included 1423 Japanese cases and 1318 controls in the discovery sample with a mixture of surgically confirmed and clinically diagnosed cases, and identified association with rs10965235 on chromosome 9p21.3 with an odds ratio (OR) of 1.44 (95% CIs: 1.30–1.59). The SNP with the lowest *P*-value or sentinel SNP rs10965235 is located in intron 6 of the cyclin-dependent kinase inhibitor 2B antisense RNA (*CDKN2BAS*). A second, smaller, Japanese GWAS with 696 patients with endometriosis and 825 controls did not find genome-wide significant associations (Adachi *et al.*, 2010).

The International Endogene Consortium (IEC) GWAS by Painter and others included 3194 surgically confirmed endometriosis cases in the discovery sample and 7060 controls of European ancestry from Australia and the UK (Painter *et al.*, 2011). Disease severity was assessed retrospectively from surgical records using the rAFS classification system and grouped into two phenotypes: Stage A (AFS stage I or II disease or some ovarian disease with a few adhesions; *n* = 1686, 52.7%) or Stage B (AFS stage III or IV disease; *n* = 1364, 42.7%; unknown *n* = 144, 4.6%) (Painter *et al.*, 2011). An important result from the IEC GWAS was that, analysing all SNPs together, it was possible to get an estimate of the genetic contribution of all common SNPs to risk of endometriosis. Overall the proportion of variation in endometriosis risk explained by common SNPs was 0.27 (s.e. = 0.04; *P* = 4.4 × 10^−16^). When this method was applied separately to the two different disease stages, genetic loading for 1364 cases with Stage B endometriosis was much greater than for 1666 cases with Stage A disease (proportion of endometriosis variation explained by common SNPs: 0.34 (s.e. = 0.04) versus 0.15 (s.e. = 0.15), respectively; *P* = 1.8 × 10^−3^). Reasons for the higher genetic loading in severe cases are not known. It may mean that there are genetic contributions to disease progression or some variants predispose directly to severe disease.

The IEC GWAS observed two genome-wide significant results, for rs1250248 on chromosome 2q35 within fibronectin 1 (*FN1*; *P* = 3.2 × 10^−8^) and rs12700667 in an intergenic region on chromosome 7 (Fig. 1). In the replication phase, 70 SNPs with nominal evidence of association were genotyped in an independent dataset comprising 2392 self-reported cases and 2271 controls of European ancestry from the US Nurses' Health Study I and II. The association on 7p15.2 with rs12700667 was replicated (*P* = 1.2 × 10^−3^). However, there was no evidence for replication of rs12540248 (*FN1*) or association with the remaining SNPs.

Published results from the Japanese and European studies only provided lists of the most significantly associated hits (*P* < 1 × 10^−5^). Comparing published data provided evidence for replication of association with rs7521902 close to wingless-type MMTV integration site family, member 4 (*WNT4*) on 1p36.12 (Painter *et al.*, 2011). Two recent replication studies genotyped top GWAS SNPs from both studies. Four variants were genotyped in 305 surgically confirmed endometriosis cases in Italy and results compared with 2710 population controls (Pagliardini *et al.*, 2013). SNPs close to *CDKN2BAS*, *WNT4* and *FN1* showed evidence of association with disease in this independent sample. The result for *FN1* is interesting because there was no evidence of replication in the US Nurses' Health Study Sample (Painter *et al.*, 2011). Analysis of 1129 surgically confirmed cases and 831 controls from Belgium (Sundqvist *et al.*, 2013) did not find significant evidence of association for either rs12700667 on chromosome 7 or for rs7521902 on chromosome 1. However, the directions of effect are consistent across all studies.

A formal multi-ethnic GWAS meta-analysis of the European and Japanese GWAS data was recently published (Nyholt *et al.*, 2012). The results demonstrated that the top hit on chromosome 7p15.2 for SNP rs12700667 did replicate in Japanese cases (*P* = 3.55 × 10^−3^, OR = 1.22) and the meta-analysis of 4604 endometriosis cases and 9393 controls provided strong evidence of association for this SNP (*P* = 9.3 × 10^−10^, OR = 1.22 (95% CI = 1.14–1.30)). A novel locus at chromosome 12q22 near the VEZT gene was identified (allele C of rs10859871: OR = 1.18, 95% CI = 1.12–1.25; *P* = 5.5 × 10^−9^). Meta-analysis also confirmed association with rs7521902 in the region of *WNT4* (*P* = 4.6 × 10^−8^, OR = 1.18, 95% CI: 1.11–1.25) and replicated association with rs13394619 near the gene growth regulation by estrogen in breast cancer 1 (*GREB1*) on chromosome 2p25.1 (*P* = 2.1 × 10^−5^, OR = 1.12, 95% CI: 1.06–1.18). *GREB1* was previously implicated with suggestive association in a small independent Japanese GWA study (Adachi *et al.*, 2010; Nyholt *et al.*, 2012).

Additional meta-analyses of the two studies excluding endometriosis cases with known minimal (Stage A) endometriosis (rAFS stage I or II disease) (American Society for Reproductive Medicine 1997) in the Australian and Oxford samples implicated a novel locus on 6p22.3 (rs7739264; *P* = 5.8 × 10^−8^, OR = 1.21, 95% CI: 1.13–1.30) close to *ID4* (subsequently replicated in the Utah study) and an independent intergenic SNP on 9p21.3 ∼55 kb from rs10965235 and 49 kb from the 3′ end of *CDKN2BAS* (rs1537377; *P* = 1.1 × 10^−8^, OR = 1.21). Polygenic prediction analysis using data from all SNPs showed significant overlap in polygenic risk of endometriosis between the European and Japanese GWA cohorts. The maximum signal (*P* = 8.81 × 10^−11^) was seen including all SNPs nominally associated with *P* < 0.1. The results suggest that many common genetic variants represent true risk variants and contribute to endometriosis risk in both populations. They also suggest that risk prediction and future targeted disease therapy may be transferred across these populations.

The most recent endometriosis GWAS was performed using 2019 surgically confirmed endometriosis cases of European ancestry from Utah and 14 471 population-based controls (Albertsen *et al.*, 2013), again providing strong replication for association in the region of *LINC00339*-WNT4 on chromosome 1p36. Furthermore, they reported novel association with rs1519761 and rs6757804 on 2q23.3 between Rho family GTPase 3 (*RND3*) and RNA-binding motif protein 43 (*RBM43*; *P* = 4.70 × 10^−8^, OR = 1.20, 95% Cl: 1.13–1.29 and *P* = 4.05 × 10^−8^, OR = 1.20, 95% CI: 1.13–1.29, respectively) in combined analysis of the discovery and replication sample (Fig. 1). They also reported suggestive association with two regions: *RNF144B*-*ID4* on chromosome 6p22.3 (rs6907340; *P* = 2.19 × 10^−7^, OR = 1.20, 95% CI: 1.12–1.28) and *HNRNPA3P1*-*LOC100130539* on chromosome 10q11.21 (rs10508881; *P* = 4.08 × 10^−7^, OR = 1.19, 95% CI: 1.11–1.27).

### Uterine fibroids

Uterine fibroids, also known as leiomyomas, are common benign tumours of the female reproductive tract. They arise in smooth muscle cells of the myometrium and although the majority are asymptomatic, they can result in pelvic pain, abnormal bleeding, infertility and pregnancy complications (Buttram and Reiter, 1981). There is evidence for a genetic component to predisposition from both familial aggregation and twin studies (Ligon and Morton, 2001). Genetic linkage studies have identified evidence for linkage to two regions on chromosomes 3p21 and 10p11 and suggestive linkage to five other regions (Eggert *et al.*, 2012). GWAS analysis in Caucasian women found genome-wide association with rs4247357 on chromosome 17q25.3 under one of the suggestive linkage peaks. This signal spans genes for fatty acid synthase (*FASN*), coiled-coil-domain (*CCDC57*) and solute carrier family 16, member 3 (*SLC16A3*). Fatty acid synthase levels were elevated 3-fold in tissue from leiomyoma tissue compared with matched myometrial tissue.

A GWAS in Japanese women from the Biobank of Japan identified novel loci associated with risk of fibroids (Cha *et al.*, 2011). The discovery sample included 1607 clinically diagnosed cases and 1428 controls and results were followed up in an additional 3466 cases and 3245 controls. Three loci on chromosomes 10q24.33 (rs7913069; *P* = 8.65 × 10^−14^, OR = 1.47), 22q13.1 (rs12484776; *P* = 2.79 × 10^−12^, OR = 1.23) and 11p15.5 (rs2280543; *P* = 3.82 × 10^−12^, OR = 1.39) showed genome-wide significant associations. There were multiple genes in each region and further functional studies will be required to determine the specific genes involved and their role in the development of fibroids.

The incidence of uterine fibroids is much greater among African American women than women of European origin. Analysis of epidemiologic risk factors for uterine fibroids in African American and women of European ancestry undergoing hysterectomy showed that the only factors statistically related to higher rates in African American women were ethnicity and no pregnancies (Moorman *et al.*, 2012). The role of genetic background was confirmed by admixture mapping with ancestry informative markers showing that the mean proportion of European ancestry was much lower in women with fibroids compared with controls (Wise *et al.*, 2012). In this study, a set of markers for loci reported in the GWAS for uterine fibroids in Japanese women was also tested, but no associations from the Japanese study replicated in the African American sample. Larger studies are required to identify more genes associated with fibroids and determine whether there are differences in the risk genes between different genetic backgrounds.

### Age at menarche and age at menopause

Age at menarche and age at natural menopause in women define the beginning and the end of reproductive life. The age for both menarche and menopause varies between individuals and between ethnic groups and is associated with a range of health conditions including breast cancer, cardiovascular disease and osteoporosis (He and Murabito, 2012). They are both complex traits influenced by a range of environmental and genetic factors. The heritability for age at menarche estimated from twin and family studies has a range from 53 to 74% and for age at menopause the range is 44–65% (He and Murabito, 2012). A number of studies have sought to identify genes associated with both traits. These have been reviewed recently (He and Murabito, 2012) including detailed summaries of the genes and regions associated with both age at menarche and age at menopause.

For age at menarche, four GWAS were published in 2009 (He *et al.*, 2009; Liu *et al.*, 2009; Perry *et al.*, 2009; Sulem *et al.*, 2009). The first studies identified novel association signals on chromosome 6q21 near lin-28 homolog B (*Caenorhabditis elegans*) (*LIN28B*) and an intergenic region on chromosome 9q31.3. Variation in the region of *LIN28B* is also associated with other markers of puberty in both boys and girls (Ong *et al.*, 2009). This region had previously been associated with adult height (Gudbjartsson *et al.*, 2008) and variation around *LIN28B* influences growth from birth to adulthood with sex-specific effects (Widen *et al.*, 2010). To increase study power, the International ReproGen Consortium was formed and expanded to conduct a meta-analysis in ∼88 000 women of European ancestry with replication in a further 15 000 women (Elks *et al.*, 2010). The original ‘hits’ were confirmed along with a further 30 novel genome-wide significant loci.

**Figure 2:**
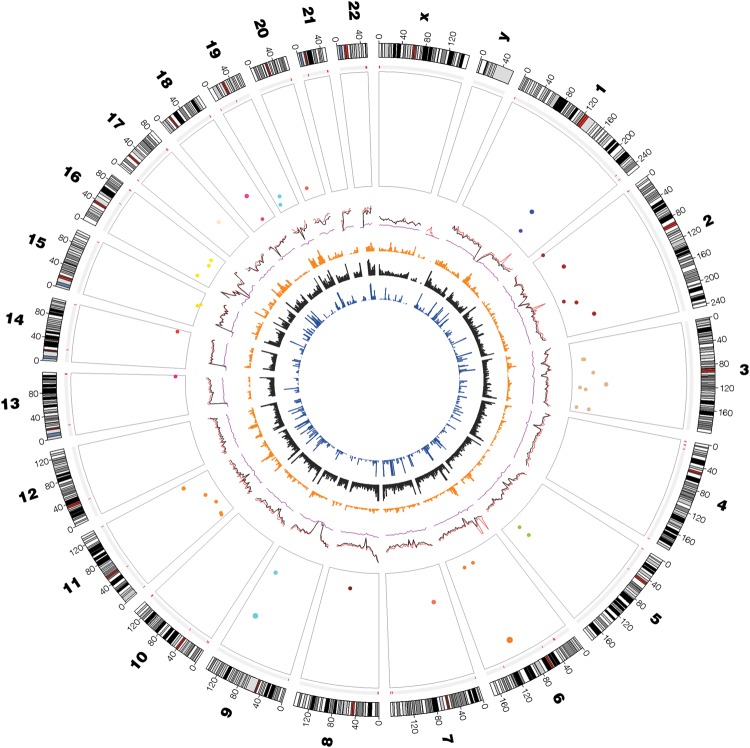
Circle plot showing the distribution of genome-wide significant SNPs (*P* < 5 × 10^−8^) and suggestive SNPs (*P* < 8 × 10^−7^) associated with age at menarche from a meta-analysis of data from 87 802 women of European descent (Elks *et al.*, 2010). The circle plot was generated using the GWASrap website (http://jjwanglab.org/gwasrap) and shows the individual chromosomes around the outside of the circle and SNPs associated with age at menarche (located in individual boxes for each chromosome). SNPs are distributed across chromosomes and across the genome. Details of the other genome features in the plot are detailed on the GWASrap website.

**Figure 3:**
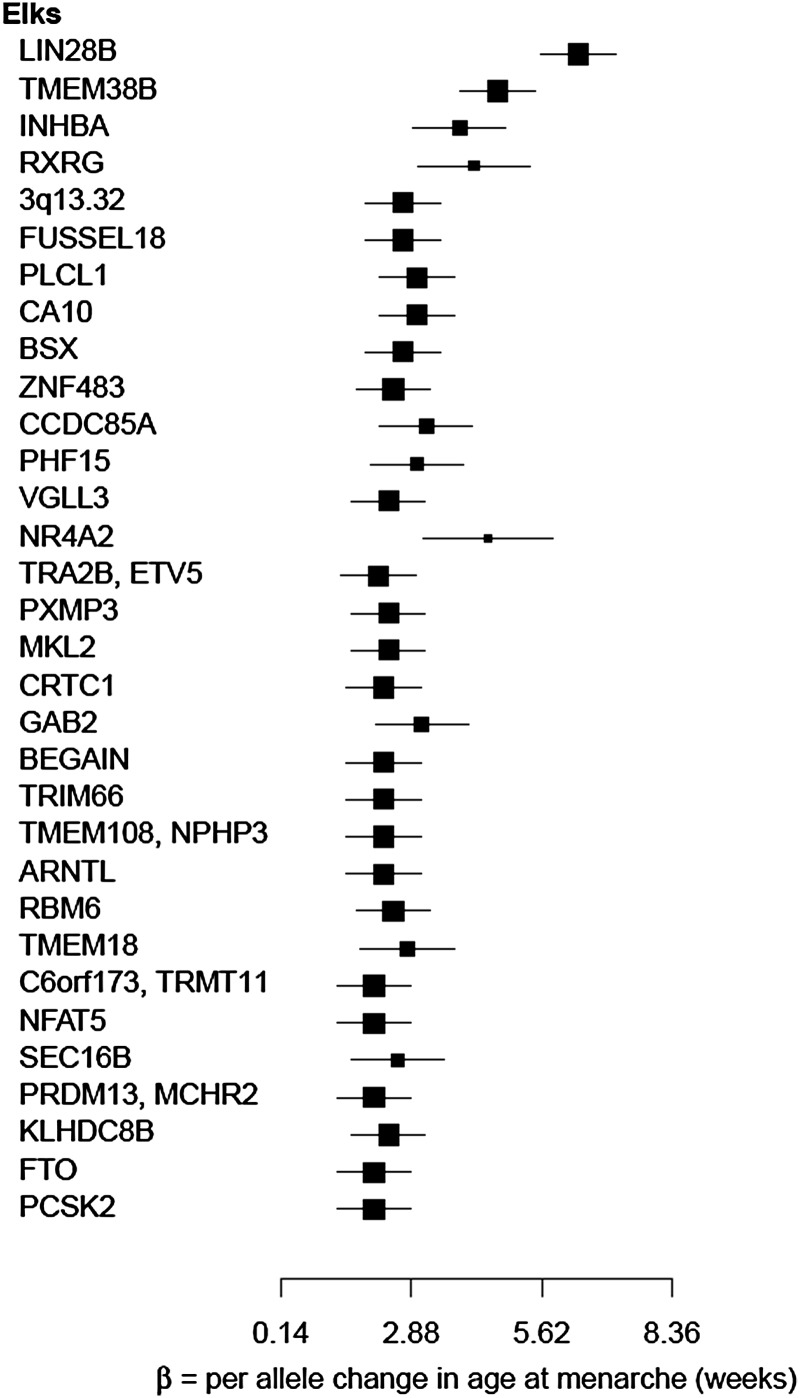
Effect size (in weeks) per allele change for individual loci associated with age at menarche from a meta-analysis of data from 87 802 women of European descent (Elks *et al.*, 2010). The effect size estimates are represented by box and whisker plots for the sentinel SNP (SNP with the lowest *P*-value) for each genome-wide significant locus. The mid-point of each box represents the point effect estimate (OR) for each locus. The width of the line shows the 95% confidence intervals of the effect estimate of individual studies.

The variants are located across multiple chromosomes (Fig. 2). Genes with greatest effects were discovered in the initial studies (Fig. 3). The large meta-analyses confirmed these ‘hits’ and identified more genes with smaller effects (Fig. 3). Overall the 42 loci account for only 4–6% of variation in age at menarche (Elks *et al.*, 2010; He and Murabito, 2012). These variants are located in or near genes from diverse pathways with the best evidence for general pathways of ‘gene expression, cellular growth and proliferation’ and ‘cellular function and maintenance’ (Elks *et al.*, 2010). Analyses of longitudinal measures linking pubertal height growth, timing of puberty and childhood obesity identified 10 loci significantly linked to pubertal growth (Cousminer *et al.*, 2013). Half of these loci were also linked to age at menarche and revealed complex genetic architecture underlying growth, timing of puberty and adiposity. Some loci showed the expected parallel association between early menarche and decreased pubertal height growth, but the T allele of rs7759938 at *LIN28B* is associated with early puberty and shorter pre-pubertal height (Cousminer *et al.*, 2013).

A recent study considered the role of markers associated with obesity and age at menarche because of the well-known inverse relationship between obesity and timing of puberty (Fernandez-Rhodes *et al.*, 2013). The study analysed 95 SNPs identified from studies of body mass index, waist circumference and waist-hip ratio for association with age at menarche in 92 105 women of European ancestry. Previously reported associations for 11 adiposity markers with age at menarche were confirmed and six novel associations of body mass index loci with age at menarche were reported. All 17 loci showed inverse relationships between BMI and age at menarche (Fernandez-Rhodes *et al.*, 2013). The authors had predicted that genetic variants associated with increased central fat were most likely to be associated with timing of menarche, but there does not appear to be strong evidence for this hypothesis.

Similar approaches have been taken to discover genes contributing to age at menopause. Two studies published GWAS data in 2009 reporting five novel regions associated with timing of menopause (He *et al.*, 2009; Stolk *et al.*, 2009). The loci were located on chromosome 5q32 in or near ubiquitin interaction motif containing 1 (*UIMC1*) and hexokinase 3 (white cell) (*HK3*), on chromosome 6p24 in synaptonemal complex protein 2-like (*SYCP2L*), on chromosome 13q34 near Rho guanine nucleotide exchange factor (GEF) 7 (*ARHGEF7*), on chromosome 19q13 in or near BR serine/threonine kinase 1 (*BRSK1*) and on chromosome 20p12.3 in minichromosome maintenance complex component 8 (*MCM8*). The International ReproGen consortium has conducted a meta-analysis of age at menopause in nearly 40 000 of European ancestry (Stolk *et al.*, 2012). Significant ‘hits’ were genotyped in a replication sample of 14 000 women. Four of the previous five loci were confirmed and 13 novel loci identified. The 17 loci account for 2.5–4.1% of the variation in age at menarche (Stolk *et al.*, 2012).

GWAS for early menopause in 3493 cases (defined as age at menopause before age 45) and 13 598 controls from 10 independent studies (with at least 100 cases) found no evidence for novel loci specifically associated with early menopause (Perry *et al.*, 2013). Four loci previously associated with normal age at menopause showed genome-wide association with early menopause and all 17 SNPs previously reported for effects on normal age at menopause showed nominal association with early menopause with the same direction of effect for both studies (Perry *et al.*, 2013). Using a polygenic model, the proportion of variation explained by all common variants captured on the SNP arrays was estimated at 21% for age at menopause as a quantitative trait (Perry *et al.*, 2013). The combined data suggest that there are multiple genes involved in determining age at normal menopause and they also play a role in early menopause. As discussed below, arrays for common variants do not capture information from rare variants and these data do not exclude a role for rare variants, sometimes with relatively large effects.

## Genetic architecture of common diseases

The genetic architecture for a disease or trait is defined as the number of loci affecting the trait, the distribution of effect sizes, interactions between the genes or loci and interactions with the environment (Stranger *et al.*, 2011). Common variants robustly associated with disease risk have been found for almost all complex diseases investigated including many diseases associated with reproduction. The discoveries provide new insights into the biology and genetic architecture of common complex diseases. In general effect sizes are small (odds ratios between 1.1 and 1.5) and the markers will not provide direct diagnostic tests. Translation of results should aid improved prevention, diagnosis and treatments, but the road to better health outcomes depends on understanding the advances and the limitations of these discoveries.

### Distribution of effect sizes

Empirical observations confirm theoretical expectation that individual variants associated with common diseases have small effects on disease risk. Across a range of diseases, the odds ratios for most effects are <1.5 (Stranger *et al.*, 2011). There are exceptions with odds ratios of 3.9 and 2.5 reported for variants of *NOD1* and *IL23R* on the risk of inflammatory bowel disease (IBD) (Barrett *et al.*, 2008) while the distribution of effect sizes for the remaining 69 variants associated with Crohn's disease ranged from 1.04 to 1.74 (Franke *et al.*, 2010). Results for most genome-wide significant ‘hits’ are at the lower end of this range as demonstrated for reproductive traits (Figs 1 and 3). For example, the odds ratios for seven variants associated with endometriosis (Fig. 1) ranged from 1.15 to 1.20 (Painter *et al.*, 2011; Nyholt *et al.*, 2012). One consequence of many genes of small effect is that large studies are required to identify variants associated with disease with any degree of certainty. The majority of early candidate gene studies were conducted on small samples that do not have sufficient power to detect true genetic associations in this range (Zondervan *et al.*, 2002) and the value of small candidate studies for gene discovery is limited.

Many GWAS projects genotype samples of 2000–3000 cases and similar numbers of controls to identify variants associated with disease risk. The numbers of variants reported in these studies vary with the genetic architecture of individual diseases (Stranger *et al.*, 2011; Visscher *et al.*, 2012). However, many more risk variants remain undiscovered (Elks *et al.*, 2010; Painter *et al.*, 2011; Nyholt *et al.*, 2012). Genotyping much bigger samples and formal meta-analyses to combine the results across different studies will detect additional risk variants. Understanding the full spectrum of common variants affecting risk will help define the pathways contributing to disease and increase the range of targets for future research and intervention studies.

### Linkage disequilibrium and estimating genotypes for all common SNPs

Patterns of common genetic variation in the human genome were characterized for a number of different ethnic groups in a major study by the International HapMap Consortium (Frazer *et al.*, 2007). Large scale SNP genotyping and sequencing studies show that the alleles of SNPs near to each other tend to be strongly correlated across individuals (Daly *et al.*, 2001; Gabriel *et al.*, 2002). The genetic correlation or linkage disequilibrium (LD) between SNPs means that there are a limited number of allele combinations or haplotypes. The patterns of LD also show organization in blocks of 100–200 Kb separated by ‘hot spots’ of recombination (Daly *et al.*, 2001; Reich *et al.*, 2002; McVean and Cardin, 2005). These interesting observations have important practical applications. They provided the basis to build haplotype maps of the human genome, construct representative SNP sets for commercial genotyping chips (such as those used in the previously mentioned GWAS studies) and to estimate or impute genotypes for all common SNPs based on the data from the genotyped sets. Genotyping arrays for GWAS generally use representative SNPs that sample most common variants. Indeed, large scale GWAS studies have been made possible because of the unravelling of the LD structure of the human genome that allows selection of representative or tagging SNPs, avoiding the need to type >10 million common SNPs.

The same LD structure makes it possible to infer or impute genotypes for an individual of most of the common SNPs in the genome. Imputation methods (Li *et al.*, 2009) infer genotypes at untyped SNPs by combining data from GWAS genotypes with a reference panel of densely genotyped or sequenced samples such as the HapMap2 data with 2.5 million SNPs (Frazer *et al.*, 2007) or the 1000 Genomes Project (1 kGP) panel based on whole genome sequencing with over 30 million SNPs (http://www.1000genomes.org/home), and provide an ‘information score’ for each SNP that allows assessment of the likely accuracy of its imputation. Imputation greatly extends the data from GWAS by evaluating association with all common SNPs. Where genotypes are imputed with reasonable certainty, there is excellent concordance between imputed variants and subsequent genotyping and stronger evidence for association can be identified for SNPs in moderate LD with the best genotyped SNP (Brown *et al.*, 2008; Nyholt *et al.*, 2012). These stronger signals can help towards identifying the likely functional variants (see below).

### Meta-analyses

Meta-analyses, combining the results of individual GWAS, have greater power and identify more disease-associated variants (Manolio, 2010). The properties of LD in the human genome and development of reliable imputation methods have greatly facilitated meta-analysis for common diseases. Different commercial chips use different sets of tag SNPs in the design. Consequently, analysis of overlapping SNPs across genotyping platforms has limitations. Imputing genotypes from a standard reference panel in each study allows data to be combined for meta-analysis across studies. Results for individual SNPs must be carefully examined and care taken to control for differences in allele frequencies between groups that could lead to false-positive associations. However, replication of association across multiple studies and population groups provides the most reliable evidence of true genetic associations (Manolio, 2010). In general, results from well-conducted individual studies that meet the stringent thresholds for genome-wide significance have been confirmed in subsequent meta-analyses of the same disease. In addition, the combined results identify many novel associations.

Meta-analyses such as those for age at menarche and menopause are often carried out within large international consortia. Early examples of very large studies combined data for measurements routinely collected in multiple studies such as height and weight or clinical phenotypes collected on many individuals (Stranger *et al.*, 2011; Visscher *et al.*, 2012). More recently, large international efforts have worked to combine datasets for common diseases with a substantial public health burden to increase power for gene discovery. For example, recent studies in breast cancer analysed genetic markers in ∼70 000 cases and controls (Ghoussaini *et al.*, 2012). The results from this and studies in other diseases demonstrate that increasing numbers of genomic regions associated with disease risk are identified as study size increases (Visscher *et al.*, 2012). In general, genes or regions with the largest effects are identified in initial studies and markers reported in subsequent studies identify genes with progressively smaller effect sizes. Nevertheless, the large meta-analyses provide insights into genetic architecture and the genes and pathways contributing to disease risk. Meta-analyses of GWAS in reproductive health are still at the ‘small’ end in terms of sample size, and increases are likely to reveal further genetic variants that could flag novel biological pathways.

## The future of genetic studies

Developments in genomics and genetics that enabled large GWAS have discovered many variants affecting risk of common diseases. The distribution of effect sizes affecting common diseases is highly skewed towards small effect sizes (Stranger *et al.*, 2011). This has led some commentators to question the value and potential of these results to transform our understanding of common diseases (see Visscher *et al.*, 2012). These are important questions. What is the value of many variants of small effect? How can results be translated into better prevention and treatment? Do small effect sizes mean we need to apply alternative approaches to understand genetic contributions to complex disease? Is there still a place for GWAS in future studies? In answering these questions and planning future studies, it is important to understand that the distribution of small effect sizes is not unexpected and agrees with theoretical models (Stranger *et al.*, 2011; Visscher *et al.*, 2012). The results reflect the underlying genetic architecture showing that genetic risk of complex diseases is due to many variants with small effects.

One proposed outcome of gene discovery is to use information from associated variants for predictions of individual disease risk. This follows from earlier studies identifying genetic mutations that are rare in the general population and predict risk of Mendelian (sub-types of) diseases with high sensitivity and specificity (for example BRCA1/2 in familial breast and ovarian cancer). This scenario contrasts with that of common variants underlying common diseases, for which effect sizes are small and the frequencies of risk alleles differ only slightly between cases and controls. Consequently, for common diseases, individual variants have little diagnostic value (Fugger *et al.*, 2012). To date, even combining results from many variants has provided limited value because only a proportion of causative loci have been identified and there are substantial environmental effects that contribute to most common diseases. In fact, useful levels of prediction may only be approached when predictors are estimated from very large samples, order(s) of magnitude greater than currently available (Dudbridge, 2013). Hence, although prediction will become more feasible as sample sizes continue to grow, the current and real translational value of gene discovery in complex diseases lies in identification of genes and biological pathways affecting disease that present new targets for intervention (Fugger *et al.*, 2012; Sanseau *et al.*, 2012). The gene regions themselves are the targets for future studies and the effects of naturally occurring variation are not a good predictor of diagnostic value or the effects of direct therapeutic interventions on target genes. Indeed therapeutic interventions or new diagnostics may be directed to other genes in a relevant pathway affecting disease risk that have no natural variants affecting disease risk.

### Gene discovery and functional biology

The goal of large scale association studies is, therefore, to identify the disease causing variants, characterize their functional effects and determine the genes and pathways responsible for disease risk. The SNPs identified in GWAS are unlikely to be the causal variants because the SNPs typed in the discovery phase were generally representative tag SNPs. The associated variants are likely picking up a signal from the causal variant(s), guilt by association and not the true culprit. As discussed above, LD patterns in the human genome helped in the gene discovery phase by allowing us to type representative tagging SNPs. Full imputation of all common variants means that the likely causal variant(s) may be in the list, but the same patterns of LD that allowed imputation make the next steps of tracking down the causal variant(s) more challenging. There will often be many common SNPs in LD with the sentinel SNP and it is difficult to determine which—if any—are the true causal variant(s).

The initial results therefore represent a starting point. In many studies, imputed SNPs have stronger signals than the best genotyped SNP. In the meta-analysis for endometriosis, rs12700667 on chromosome 7 remained the sentinel SNP after imputation, but imputed SNPs at the 5′ end of *WNT4* had stronger signals than the best signal from the GWAS (rs7521902) located 20 kb upstream of the *WNT4* (Nyholt *et al.*, 2012; Albertsen *et al.*, 2013). The next important step for individual regions is to identify the specific genes and pathways implicated in disease risk. The functional variants are most likely to regulate gene expression as >80% of GWAS ‘hits’ are located in introns or within intergenic regions (Dunham *et al.*, 2012). At present, there is no definitive database to look up a set of SNPs and determine which SNP(s) is most likely to have functional effects.

One approach is to look for allele-specific differences in expression of genes or individual transcripts in the region. A number of GWAS have been conducted on mRNA expression levels, which are themselves quantitative traits (Stranger *et al.*, 2011). Genetic differences contributing to variation in gene expression are known as expression quantitative trait loci (eQTLs). Several studies show that complex trait-associated variants overlap with eQTL variants (Stranger *et al.*, 2011). The eQTLs can be close to the gene affected (*cis* effects) or the SNPs can affect gene expression at remote points on the same or different chromosomes (*trans* effects). The power to detect *trans*-eQTLs is much lower than for *cis*-eQTLs (partly because they are likely to have smaller effect sizes, and partly because of the need to adjust for the many more statistical tests conducted in *trans* analyses), and few studies have sufficient power to detect such *trans* effects. A recent eQTL meta-analysis identified and replicated *trans*-eQTLs for 233 SNPs previously associated with complex traits at genome-wide significance (Westra *et al.*, 2013). Some SNPs influenced multiple *trans*-genes. These results support the view that disease-associated variants identified by GWAS can function through effects on transcription of both closely related genes and genes on other chromosomes. Some eQTL datasets are publically available (e.g. http://www.sanger.ac.uk/resources/software/genevar/). However, many available datasets may not be relevant to diseases and traits associated with reproduction and there is a need to develop eQTL datasets for relevant tissues like the endometrium.

The international ENCODE project has made major advances in better understanding genome regulation through a systematic approach to characterizing functional elements in the genome (Dunham *et al.*, 2012). A recent series of important papers report results of systematic mapping of regions of transcription, transcription factor-binding sites, chromatin structure and histone modification in a range of cell lines (Dunham *et al.*, 2012). The results demonstrate that a large proportion of the non-coding region of the genome (introns and intergenic sequences) contain regulatory elements. These data are available in genome browsers and can be used to search for the overlap between disease-associated variants and functional elements to prioritize SNPs and genes or SNPs for follow-up functional studies. Analysis of data from the GWAS catalogue (Hindorff *et al.*, 2009) and ENCODE data shows significant enrichment of transcription factor-binding sites and DNAse I hypersensitive sites at SNPs associated with complex diseases when compared with SNPs from the rest of the genome (Dunham *et al.*, 2012). Although extensive, the complete datasets are only available for a limited number of cell lines. Identifying the functional variants for reproductive traits will require better understanding of tissue-specific gene regulation and changes in the regulation during development. One important direction for future studies in reproduction is to conduct genomic experiments in relevant cell types and tissues to identify eQTLs, map functional elements and better characterize gene regulation in tissues relevant to reproductive activity and fertility.

### Rare or low-frequency variants

Until recently, commercial genotyping chips were designed to genotype representative ‘tagging’ SNPs that captured most common variation in the genome. Analyses generally included SNPs with a minor allele frequency (MAF) of >5%. As discussed above, the tagging SNPs allowed imputation of most common SNPs in the genome. Although imputation using recent 1 kGP panels can impute a considerable proportion of SNPs with MAF < 5% (Howie *et al.*, 2012; Sung *et al.*, 2012), imputation works far better for variants with MAF > 5%. For example, of 11.5 million SNPs present in the European panel consisting of 566 haplotypes from 1 kGP data (August 2010 release), Sung and colleagues were able to successfully impute (*R*^2^ > 0.3) 0.39 million (11%) of 3.65 million SNPs with rare (MAF ≤ 0.01) and 1.23 million (55%) of 2.25 million SNPs with low frequency (0.01 ≤ MAF ≤ 0.05), compared with 5.12 million (92%) of 5.56 million SNPs with common frequency (MAF > 0.05).

Many important variants in the genome have MAFs <5%. For example, the majority of SNPs in the coding regions of genes that change the amino acid composition of the protein, alter mRNA splicing or change stop signals have MAF < 5% (Huyghe *et al.*, 2013). Given that common SNPs do not generally tag rare genetic variation, it is highly likely that the common GWAS signals significantly associated with complex diseases are not due to functional coding variants; however, current GWAS designs will have missed low-frequency coding variants (and other low-frequency functional variants) contributing to disease risk.

There is increasing evidence that low-frequency variants (LFVs) do contribute to disease risk. An LFV in the melanocyte master regulator microphthalmia-associated transcription factor (*MITF*) increases melanoma risk (OR 2.19, 95% CI 1.41–3.45) and has a large effect in individuals with family history and multiple melanomas (OR 8.37, 95% CI 2.58–23.80) (Yokoyama *et al.*, 2011). Next-generation sequencing in pooled samples from patients with Crohn's disease and controls identified additional independent risk variants in two of the known risk genes (*NOD2* and *IL23R*), a highly significant association with a protective splice variant in *CARD9* (*P* < 1 × 10^−16^, odds ratio ∼0.29), and additional associations with coding variants in several genes (*IL18RAP*, *CUL2*, *C1orf106*, *PTPN22* and *MUC19*) (Rivas *et al.*, 2011).

**Figure 4:**
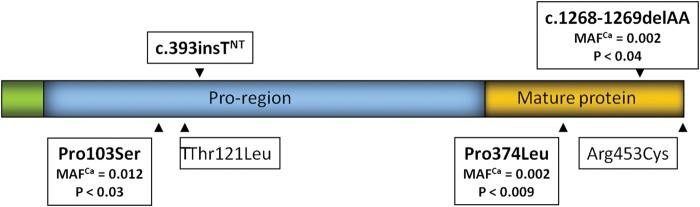
Low-frequency coding variants (LFCVs) in growth differentiation factor 9 (*GDF9*) identified by DNA sequencing in samples from mothers of dizygotic twins. The locations of LFCVs significantly associated with twinning are shown together with the minor allele frequency (MAF) in cases (Ca) and the *P*-value for association. The c.393insT LFCV was not tested (NT) in the full sample (Palmer *et al.*, 2006).

Growth differentiation factor 9 (*GDF9*) and bone morphogenetic protein 15 (*BMP15*) are expressed in oocytes and play critical roles in the regulation of ovarian follicle development. A large number of mutations in *BMP15* and *GDF9* increase the frequency of twins in sheep (McNatty *et al.*, 2004; Moore *et al.*, 2004). Sequencing the coding region of *GDF9* in women from families with a high frequency of dizygotic twins identified novel LFVs that change amino acid composition, or introduce premature stop codons (Fig. 4), and increase the risk of dizygotic twining in women (Montgomery *et al.*, 2004; Palmer *et al.*, 2006). The LFVs in *GDF9* associated with increased dizygotic twinning included two premature stop codons and two mis-sense variants, ranged in frequency from 0.002 to 0.12 and together explained about 2% of the estimated genetic variation in dizygotic twinning (Montgomery *et al.*, 2004; Palmer *et al.*, 2006).

Power for gene discovery is partly a function of allele frequency and larger samples are required to identify LFVs contributing to disease risk. Recently, low-cost commercial genotyping arrays have become available with assays for >90% of all well-documented mis-sense coding variants in the human genome (Huyghe *et al.*, 2013). The content for this chip was designed from exome sequencing of >12 000 individuals (∼10 000 of European ancestry). Non-synonymous variants were included in the design if they were observed three or more times and in two datasets, and the design captures 97–98% of non-synonymous variants and 94–95% of stop or splice altering variants in the average genome. Genotyping chips with this exome content provide a rapid, low-cost method to genotype most variants in protein coding regions in a large number of individuals.

Disease-related variants in exons that change protein composition through amino acid substitutions alter stop signals or splicing will provide direct evidence of the specific genes contributing to disease risk. Variants may also indicate the likely functional consequences of the altered proteins. The fine mapping and functional studies required to determine the specific genes affected by common non-coding variants will not be necessary. Consequently, low-frequency coding variants could provide a more direct path to develop more effective preventative and therapeutic strategies.

### DNA sequencing studies

Major advances in sequencing technology have broad applications in genetics and genomics. Sequencing can speed up identification of causal variants in rare Mendelian disease, help understand the functional role of genetic variation and facilitate discovery of further disease-associated variants. Examples include continued discovery of common and rare variants through sequencing (Manolio, 2013) and the wide application of DNA sequencing in the ENCODE project to identify functional regions of the genome (Dunham *et al.*, 2012). As sequencing costs fall, some commentators have suggested that GWAS will largely be replaced by sequencing. However, the cost of genotyping remains cheaper than sequencing and the challenges and cost of analysis for genotype calling in sequence data limit the applications of sequencing. As we seek to understand the role of LFVs, very large samples must be studied and projects can be conducted on a much larger scale with current genotyping technology. The future of gene-mapping studies is likely to see the parallel use of sequencing and genotyping for continued discovery of disease-associated variants.

### The role of large studies with detailed phenotypic data

The number of discovered variants is strongly correlated with experimental sample size, where an ever-increasing sample size will increase the number of discovered variants (Visscher *et al.,* 2012). International efforts combining results of many studies in big meta-analyses have been the best approach to gene discovery for common diseases. This has been most successful for universal traits like height and weight because these traits were measured on many cohorts and sample sizes of ∼190 000 have been included in some studies (Lango Allen *et al.*, 2010). In contrast, efforts for many reproductive diseases are based on modest samples, and have therefore detected only a small number of significant associations. Important exceptions include traits such as age at menarche that has been measured in many studies or breast cancer where large international consortia studying different aspects of breast cancer have actively recruited patients and combined studies to greatly increase the size of studies for gene discovery. Some argue that further studies show diminishing returns and we expect that effect sizes of subsequent discoveries will be smaller. However, effect size for an individual variant does not reflect the importance of the pathway to the disease or ability to develop diagnostic or therapeutic outcomes. Discovery of additional variants increases the chances for finding tractable targets for immediate follow-up and clinical outcomes.

Therefore, diseases with a genetic component associated with reproduction will continue to benefit from additional GWAS studies and large meta-analyses to define more of the genetic variants that contribute to disease risk. Discovery of LFVs associated with disease by genotyping exome chips or by high throughput sequencing will help understand functional pathways leading to disease. However, many of the current large collections have limited phenotype and clinical information. Small clinic-based samples with detailed information on individual patients are not large enough for gene discovery and larger sample collections generally lack detailed information on treatments and risk factors. The large meta-analyses combine data sets where disease phenotypes and risk factors may have been recorded in different ways. Differences in disease definition are likely to be important and averaging across studies with different methods of ascertaining disease cases may lead to under-estimation of effect size for some variants. Consequently, the results from GWAS data are limited by the minimal phenotypic and clinical information collected for most sample sets. Phenotypic measurements are expensive and it will be a challenge to generate these rich datasets. Although small clinic-based samples with detailed information on individual patients may prove useful in examining genotype–phenotype correlations for variants implicated by large GWAS meta-analyses, harmonization of phenotypic data collection in different centres, along with protocols for biological sample collection, is an important next step that will facilitate both novel discovery and translational follow-up of genetic results (see, for example, the WERF EPHect initiative in endometriosis: http://endometriosisfoundation.org/ephect/). Combination of datasets with detailed harmonized phenotypic and clinical information combined with current genomics tools will yield valuable insights into disease risks, disease classification and co-morbidity.

## Translation of GWAS results to the clinic

Gene discoveries from GWAS do not generally provide results that can be translated immediately into the clinic. They are the starting point to understand disease biology and have already provided novel insights into the pathogenesis of several diseases. Variants that increase the risk of type 2 diabetes influence beta-cell development and function and focus attention on insulin secretion in the development of disease (Grarup *et al.*, 2010). Discoveries in IBD have highlighted the importance of the autophagy pathway in disease development (Rioux *et al.*, 2007; Xavier *et al.*, 2008). Results for endometriosis suggest effects on estrogen response and cell growth rather than inflammation (Nyholt *et al.*, 2012). Genetic variants in the interleukin 23 and interleukin 17 pathways are associated with susceptibility to psoriasis suggesting that targeting this pathway might have therapeutic benefit. Monoclonal antibodies neutralizing these genes have been shown to be effective in treating psoriasis and several compounds targeting this pathway are in clinical development (Fugger *et al.*, 2012; Gudjonsson *et al.*, 2012).

### Genotype–phenotype relationships

Genetic and environmental factors both influence the risk of complex diseases and understanding environmental risk factors has also proved difficult. The advances in gene discovery will be useful in defining some of these environmental risk factors. One example is studies on the role of autophagy related 16-like 1 (*Saccharomyces cerevisiae*) (*ATG16L1*) in the risk of Crohn's disease. A knock down of Atg16l1 in mice induces a phenotype similar, but not identical, to Crohn's disease (Cadwell *et al.*, 2010). Mice raised in a specific pathogen free environment do not have the phenotype, but symptoms return in the presence of a specific mouse norovirus (Cadwell *et al.*, 2010). In endometriosis there are suggestions of effects of environmental toxins and interactions with genotype, but the topic remains controversial (Pauwels *et al.*, 2001; Reddy *et al.*, 2006; Trabert *et al.*, 2010; Vichi *et al.*, 2012). Well-designed genotype × environment studies may help identify the role of important environmental factors and suggest treatments or lifestyle changes that can minimize disease risk for some individuals.

### New drug targets

Gene discovery has occurred rapidly over the last 5 years and immediate translation of these discoveries is not realistic. The biological insights into disease risk factors do provide new drug targets. However, development and testing of new drugs can take many years. One approach is drug repositioning through the analysis of GWAS results to identify alternative indications for existing drugs (Sanseau *et al.*, 2012). Data from the published GWAS catalogue were used to construct a list of GWAS genes associated with disease traits and investigate whether these genes are targeted by drugs already launched or in development. The results showed that of 991 genes considered, 21% were considered ‘drugable’ by small molecules and 47% potentially targeted by therapeutic antibodies or protein therapeutics (Sanseau *et al.*, 2012). These proportions were significantly higher than genes across the genome. Moreover, 155 of the 991 genes implicated from GWAS (15.6%) have an associated drug project already in the pipeline. This is 2.7 times higher than for all genes in the genome and more than expected by chance. Examples include well-validated targets and associated drugs such as 3-hydroxy-3-mehtylglutaryl-CoA reductase (*HMGCR*), the target for statins lowering cholesterol (Sanseau *et al.*, 2012). This analysis of drug repositioning highlights the power of GWAS studies in defining new drug targets and providing biological insights to help streamline drug development.

### Applications of GWAS data beyond the top hits

Genetic profiles can also be used in important ways to investigate genetic co-morbidity and to evaluate use of current diagnostic criteria in closely related disease conditions. We have shown for endometriosis (Painter *et al.*, 2011; Nyholt *et al.*, 2012) that analyses of all SNPs in GWAS data sets provide powerful approaches to investigate subgroups within disease phenotypes and understand shared genetic contributions across studies. Association results must pass stringent thresholds for significance and be replicated in independent studies before risk variants are accepted as contributing to disease risk. Only a few of the top hits meet these criteria in most genome-wide studies. However, many other variants lie just below the threshold. Some of these will be ‘truly’ associated with disease, but cannot be distinguished from the other false-positive signals. Larger studies help to discover more of the risk variants, but the application of multivariate statistical approaches to the entire marker dataset can be used in other important ways to understand the nature of genetic contributions to disease risk.

It is often difficult to determine the relationship between disease classes with strongly overlapping symptoms. In genetic studies of endometriosis, the Revised American Fertility Society (rAFS) classification system is commonly used to stage disease severity and assigns patients to one of four stages (I–IV) on the basis of the extent of the disease and the associated adhesions present (Montgomery *et al.*, 2008; Rahmioglu *et al.*, 2012). Other classification systems have been proposed including ovarian versus peritoneal disease, and deep infiltrating versus superficial disease. Whether these sub-classes represent the natural history of one disorder, or are in fact different disease sub-types, is an important consideration in endometriosis research. Analysis of genome-wide marker data can assess the genetic contribution to individual disease sub-classes and also the shared genetic contribution to each subclass and provide new insights into the different disease presentations. Large samples with detailed data on symptoms and disease classification will facilitate these studies and may provide important insights for future diagnosis and treatment.

Another approach is to use genome marker data to evaluate comorbidity between disease conditions. Epidemiological studies can be difficult to interpret because there may be problems with ascertainment and large cohorts must be recruited to have sufficient numbers of patients with both conditions to enable firm conclusions to be drawn. For example, investigating co-morbidity of ovarian cancer or endometrial cancer with endometriosis is problematic because of the potential incidental diagnosis of endometriosis at laparoscopy as part of investigations of symptoms for ovarian or endometrial cancer. The advent of genome-wide marker data offers an alternative approach by evaluating shared genetic contributions to disease traits directly using the GWAS genotypes. Epidemiological evidence also suggests comorbidity between schizophrenia and cardiovascular disease. Leveraging the large GWAS studies conducted on cardiovascular disease identified additional loci associated with schizophrenia (Andreassen *et al.*, 2013). The overlap in genetic risk is important since there is significant mortality from cardiovascular disease in patients with schizophrenia suggesting the need to better monitor cardiovascular disease in these patients (Gegenava and Kavtaradze, 2006; Laursen *et al.*, 2012). Analysis of GWAS data across disease studies can lead to a better understanding of the shared genetic contributions to disease and possible re-assessment of diagnostic criteria. This could be an important avenue for translation of genomics to improve clinical practice.

## Summary and conclusion

Genome-wide association studies provide a powerful approach for the discovery of genes or variants contributing to risk of complex diseases. Results for multiple traits and diseases are reported in over 1600 publications and documented in the Catalog of Published Genome-Wide Association Studies at the National Human Genome Research Institute. Included in these studies are results for over 30 traits and diseases related to reproduction documenting many novel findings. Results generally show that genetic contributions to complex disease come from many gene regions across the genome, each with small effects on disease risk. Consequently, studies on large samples are essential to identify the many individual variants affecting disease risk. Combined studies have been undertaken for traits like age at menarche, breast cancer and prostate cancer. However, most studies for diseases associated with reproduction have been relatively small. Results show genetic data can also help define sub-types of disease and co-morbidity with other traits and diseases. Consequently, future genetic marker studies in large samples with detailed phenotypic and clinical information will yield valuable insights into disease risks, disease classification and co-morbidity for many diseases associated with reproduction.

The value of GWAS has been questioned by some commentators because variants discovered have such small effects. Even when combined, the small numbers of variants identified thus far have little diagnostic value for individuals because of their small effects coupled with environmental influences. However, the real translational value of gene discovery in complex traits lies in discovery of genes and biological pathways affecting disease that present new targets for intervention. The initial results therefore represent a starting point, and for diseases like endometriosis, the first step in defining causal pathways to disease. Much work remains to determine the mechanisms in each defined region. Eighty per cent of markers associated with common disease lie in intronic and intergenic regions with no easy functional explanation for increased disease risk. Indeed, GWAS studies have revealed how much we still have to learn about the control of gene transcription. Genomic studies such as the ENCODE project are helping to fill the gap and as functional studies progress, laboratories with specialized knowledge of specific genes and pathways can help unravel important mechanisms leading to disease. Studies of rare coding variants affecting risk may also help.

Novel genes and pathways provide new targets for biomarker discovery and new drug targets for drug development or repositioning of drugs currently on the market or in clinical trials. Genetic variants can help understand important environmental risk factors for targeted intervention. Genetic studies have much to contribute to future studies in reproduction. However, the real benefits will only be realized by convergence of genetics, genomics and biological research in well-phenotyped datasets to develop better methods of diagnosis and treatment for the many common diseases associated with reproduction.

## Authors' roles

G.W.M., K.T.Z. and D.R.N.: conception and design, drafting manuscript, revising manuscript for critical comment and final approval of manuscript.

## Funding

G.W.M. was supported by the NHMRC Fellowship scheme (339446 and 619667). D.R.N. was supported by the NHMRC Fellowship (339462 and 613674) and Australian Research Council (ARC) Future Fellowship (FT0991022) schemes. K.T.Z. is supported by a Wellcome Trust Career Development Award (WT085235/Z/08/Z). Funding to pay the Open Access publication charges for this article was provided by the Wellcome Trust.

## Conflict of interest

None declared.
